# Genomic presence of recombinant porcine endogenous retrovirus in transmitting miniature swine

**DOI:** 10.1186/1743-422X-3-91

**Published:** 2006-11-02

**Authors:** Stanley I Martin, Robert Wilkinson, Jay A Fishman

**Affiliations:** 1 Infectious Disease Division, Massachusetts General Hospital, Boston, MA 02114, USA

## Abstract

The replication of porcine endogenous retrovirus (PERV) in human cell lines suggests a potential infectious risk in xenotransplantation. PERV isolated from human cells following cocultivation with porcine peripheral blood mononuclear cells is a recombinant of PERV-A and PERV-C. We describe two different recombinant PERV-AC sequences in the cellular DNA of some transmitting miniature swine. This is the first evidence of PERV-AC recombinant virus in porcine genomic DNA that may have resulted from autoinfection following exogenous viral recombination. Infectious risk in xenotransplantation will be defined by the activity of PERV loci in vivo.

## Background

Xenotransplantation using inbred miniature swine is a proposed solution to the shortage of organs available for transplantation. Three subgroups of porcine endogenous retroviruses (PERV) have been identified, PERV-A, -B, and -C. PERV-A and PERV-B have been shown to replicate in human cells in vitro, while PERV-C is largely restricted to porcine cells [1-5]. Infection of human subjects has not been identified in individuals with exposure to porcine tissue [3,6-9], though concern about the risk of cross-species infection in xenotransplantation still exists.

It has been demonstrated that PERV replicating efficiently in human cells in vitro is a recombinant of PERV-A and -C within the *env *region, and probably arises from exogenous recombination of mRNA [2,5,10]. Following cocultivation of "transmitting" porcine peripheral blood mononuclear cells (PBMC) with human cell lines, PERV-AC recombinants have been identified within human cells in vitro [2,4,5]. PERV-AC recombinant provirus has not been detected previously in the genomes of transmitting swine [5,10].

To examine the mechanisms underlying PERV recombination, four animals not known to transmit recombinant virus to human cells in vitro and four animals previously characterized as having a transmitting phenotype were identified from a herd of inbred miniature swine [5,11]. Polymerase chain reaction (PCR) assays of tissue samples and coculture studies between porcine and human cells were undertaken to better characterize recombinant PERV in vivo.

## Results

**Figure 1:**
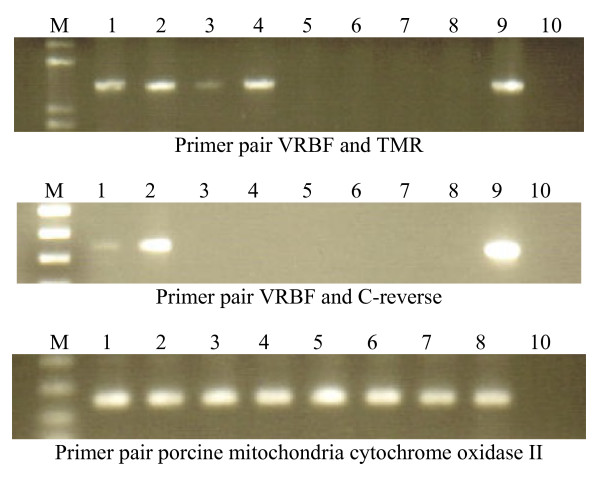
Gel electrophoresis from PCR of genomic DNA from multiple samples. Lane M represents the lane marker. Samples are as follows: 1, 15149; 2, 13910; 3, 13653; 4, 15150; 5, 15578; 6, 15579; 7, 12910; 8, 16181; 9, A14-220; and 10, water. The A14-220 DNA serves as a positive control for PCR involving recombinant PERV-AC, the water as a negative control. Bands seen with primer pairs VRBF and TMR are between the 1000 and 1650 base pair lane markers. Bands seen with primer pairs VRBF and C-reverse are between the 300 and 400 base pair lane markers.

**Figure 2:**
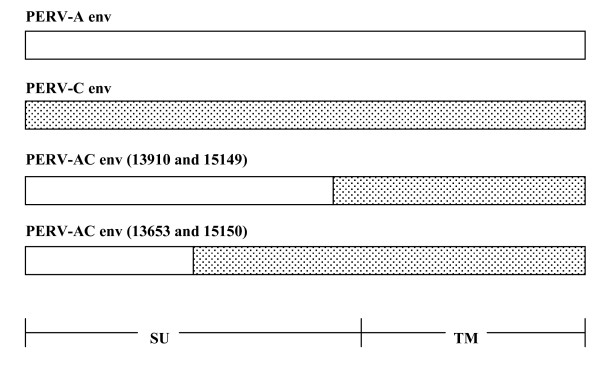
Diagram illustration of the PERV-AC envelope generated by primer pair VRBF and TMR from animals 13910, 15149, 13653, and 15150, compared to envelopes from PERV-A (clear) and PERV-C (shaded).

**Figure 3:**
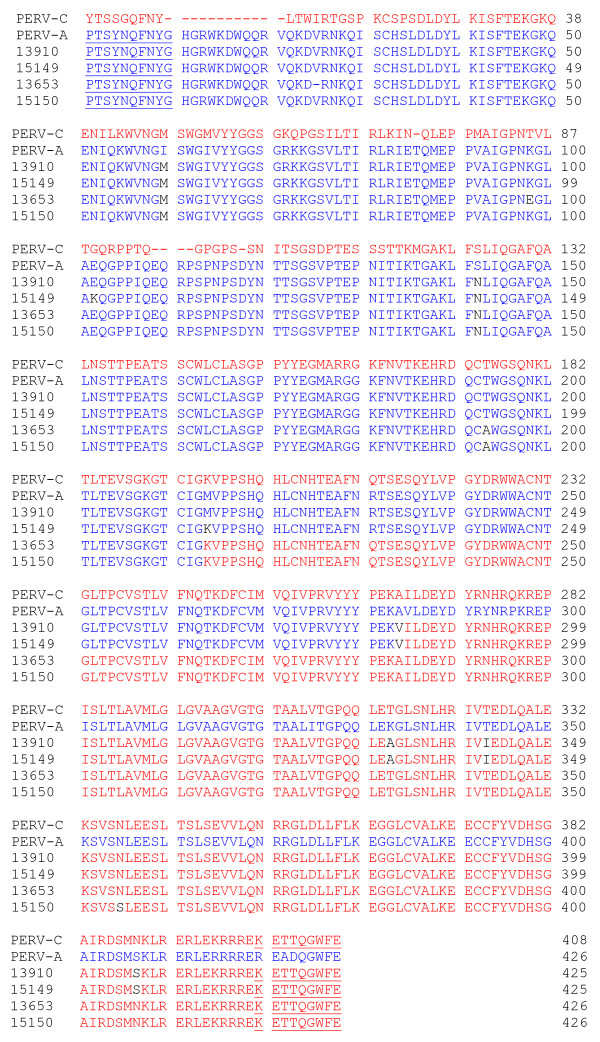
Deduced PERV-AC amino acid sequences from all four transmitters are shown in alignment with a PERV-A and PERV-C *env *sequences. Amino acid 279 from PERV-C, 297 from PERV-A, 296 from subjects 13910 and 15149, and 297 from subjects 13653 and 15150, represent the start of the TM region of the *env*. Blue amino acids represent PERV-A and red amino acids represent PERV-C. The sequences encoded by the forward PERV-A primer (VRBF) and reverse PERV-C primer (TMR) are underlined.

Using VRBF and TMR PCR primers, PERV-AC recombinant virus was detected in the cellular DNA isolated from all four transmitting animals, 13910, 15149, 13653, and 15150 (Fig. 1). Each PCR was repeated at least three different times. Lung, heart, thymus, PBMC, abdominal and thoracic lymph nodes, spleen, liver, and kidney from animals 13910 and 15149 contained recombinant virus. Pancreatic tissue did not contain virus. Only PBMC were available for testing from animals 13653 and 15150. Sequencing performed in duplicate on samples from animals 13910 and 15149 revealed 98% homology with a previously published PERV-AC sequence from infected human cells lines, **AY364236.1**(Fig. 2 and 3) [10]. Samples from animals 13653 and 15150 showed closer homology to another PERV-AC clone, **AF417230**(Fig. 2 and 3) [2].

Comparison of sequences from 13653 and 15150 revealed a 0.2% difference (3 out of 1284 base pairs). Comparison of sequences from 3 different tissues (spleen, liver, and PBMC in 13910 and spleen and PBMC in 15149) showed a difference of 0.4 – 1.2%. Some of these minute differences within multiple samples of the same tissues are within the realm of PCR and sequencing artifact, though the presence of actual small variations cannot be ruled out. Non-transmitting swine 15578, 15579, 16181, and 12190 revealed no evidence of genomic DNA PERV-AC recombinant virus in lymph node, liver, spleen, lung, thymus, or PBMC (animals 15578 and 15579) or just PBMC (12190).

**Figure 4:**
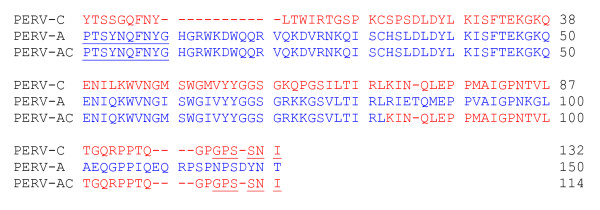
Deduced PERV-AC amino acid sequence from 13910 shown in alignment with a PERV-A and PERV-C *env *sequence. Sequences from both 13910 and 15149 were identical, hence only one representation shown. Blue amino acids represent PERV-A and red amino acids represent PERV-C. The sequences encoded by the forward PERV-A primer (VRBF) and reverse PERV-C primer (C-reverse) are underlined.

Using primer pair VRBF and PERV-C reverse, a second PERV-AC recombinant was detected in the DNA of 13910 and 15149 in all tissues tested, again with the exception of pancreas (Fig. 1). Sequencing of amplified product from PBMC of both subjects demonstrated identical 345 base pair sequences (Fig. 4). The first 254 bases had 99% homology to a sequence from the SU region of a previously published PERV-A clone DD8a8 *env *gene [2]. The remaining 91 base pairs shared 98% homology to 6 different previously published PERV-C *env *genes (**AY570980**, **AF417227**, **AF402662**, **AF402661**, **AF038600**, and **AF038599**). This same primer pair revealed no evidence of recombinant virus within the isolated DNA of cells from lymph node, liver, lung, thymus, spleen, and PBMC from swine 15578 and 15579. Only PBMC were available for testing in animals 16181 and 12190.

## Discussion

These studies demonstrate the presence of recombinant PERV-AC in the DNA of four different transmitting swine. It is unclear whether the "transmitting" phenotype is a stable or intermittent trait and whether genomic PERV-AC sites are active. Wood et al. detected PERV-AC viral mRNA in unstimulated PBMC of transmitting animals; the sequences of these PERV recombinants were not compared with those derived from infected human cells [5]. While the possibility exists that blood-derived elements account for the provirus we have detected in multiple tissues, previous studies did not demonstrate PERV-AC from PBMC of limited numbers of transmitting animals [5,10].

Multiple PERV-AC recombinants have been described after in vitro infection of human cells. Episodic viral recombination might lead to the development of multiple strains of PERV-AC in a single animal, with autoinfection and with variable integration into cellular DNA of different tissues. The absence of virus from a single tissue (pancreas) in a transmitting animal with genomic PERV-AC is consistent with this hypothesis. However, enzymatic degradation of cellular DNA and RNA in pancreatic tissue or other factors might limit detection of PERV-AC in this organ. Of note, though, smaller fragments of control DNA from the cellular DNA harvested from pancreatic samples were still amplifiable by PCR (data not shown). The presence of two different types of PERV-AC in the cellular DNA of some transmitting animals suggests, however, that PERV recombination is intermittent and leaves behind genomic evidence of autoinfectious cycles.

The characterization of transmitting or non-transmitting phenotypes may not be permanent. This idea is consistent with the suggestion that the activity of PERV-C sites "drives" the recombination process [5]. Further investigation is required to determine whether recombinant PERV can be derived from proviral DNA sites as well as from exogenous recombination of mRNA. In preliminary studies using stimulated PBMC from swine 15149 (transmitter) and 16181 (non-transmitter), transmission assays have not revealed infection of human 293 cells in vitro over 110 days of cocultivation. While the levels of PERV expression in these studies might be below the limit of detection, it is possible that the two genomic PERV-AC sequences detected here represent dysfunctional proviral elements.

The presence of PERV-AC in the DNA of porcine tissues from animals capable of transmitting infection to human cells combined with the early coculture assays sited above, suggest that the transmitting phenotype is intermittent. It remains to be determined whether the transmitting phenotype is inherited or acquired de novo in swine. It remains necessary to determine the conditions that determine the production of PERV-AC in vivo to determine the level of risk for infection of human recipients of porcine xenografts.

## Materials and methods

Tissues were harvested from each animal, including blood, heart, lung, thymus, pancreas, liver, kidney, spleen, abdominal and thoracic lymph nodes, and skeletal muscle. Swine 15578, 15579, and 16181 were bred with a deletion of the gene encoding alpha-1, 3-galactosyltransferase and are considered non-transmitters. Cell samples from animal 12190 showed no transmission to either human or porcine cells in vitro, designating it a "null transmitter" [5]. Care of animals was in accordance with the Guide for the Care and Use of Laboratory Animals prepared by the National Institutes of Health under a protocol approved by the Massachusetts General Hospital Subcommittee on Research Animal Care.

Tissues were stored at -80°C. DNA was isolated using the Puregene^® ^DNA purification protocol (Gentra Systems, Minneapolis, MN) per manufacturer's instructions. For PCR amplification, PERV-A *env *primer VRBF (forward) (5'-CCTACCAGTTATAATCAATTTAATTATGGC-3') and two different PERV-C *env *reverse primers were used: TMR (5'-CTCAAACCACCCTTGAGTAGTTTCC-3') and PERV-C reverse (5'-TATGTTAGAGGATGGTCCTGGTC-3') as previously described [5,12]. The presence of amplifiable porcine mitochondria cytochrome oxidase II was used as a DNA quality control. Forward primer (5'-TCACCCATCATAGAAGAACTCCTACA-3'), and reverse primer (5'-TTTTACGGTTAAGGCTGGGTTATTAATT-3') were used to amplify the mitochondrial DNA. Each PCR reaction involved 100 ng of isolated DNA. All PERV reactions were performed on a GeneAmp^® ^PCR system 9700 (PE Applied Biosystems, Foster City, CA) with the following parameters: 94°C for 10 minutes, 40 cycles of 94°C for 30 seconds, 55°C for 30 seconds, and 72°C for 120 seconds, followed by 72°C for 10 minutes. For detection of porcine mitochondrial markers, an annealing/melting temperature of 60°C was used.

PCR products were isolated and cloned using the *E. coli *plasmid vector pCR4-TOPO^® ^or pCR-XL-TOPO^® ^(Invitrogen, Carlsbad, CA) per the manufacturer's instructions. Plasmids were isolated using QIAprep^® ^Spin Miniprep columns (Qiagen, Valencia, CA). Sequencing was done with Applied Biosystems Taq DyeDeoxy Terminator cycle sequencing kits on an ABI 3700 PRISM automated sequencer (Massachusetts General Hospital DNA Core Sequencing facility).

Coculture assays were done using viable PBMC from animals 15149 and 16181.

PBMC were stimulated for 5 days in phytohemagglutinin and phorbol 12-myristate 13-acetate and then placed in tissue culture flasks together with target human 293 cells. Recombinant PERV-AC infection of human 293 cells was assayed using the PCR methods noted above on harvested cellular DNA from the human 293 cells on a once weekly, to once every two week basis.

## Authors' contributions

SM performed the PCR assays, cloning experiments, and sequencing analysis, as well as drafted the manuscript. RW carried out the majority of tissue and sample collection and oversaw the coculture experiments. JF conceived of the study, participated in its design and coordination, as well as made contributions to the manuscript. All authors read and approved the final manuscript.
